# Failure to identify modifiers of *NEBULIN*-related nemaline myopathy in two pre-clinical models of the disease

**DOI:** 10.1242/bio.044867

**Published:** 2019-09-17

**Authors:** Boyang Qiu, Julie Ruston, Henk Granzier, Monica J. Justice, James J. Dowling

**Affiliations:** 1 Program for Genetics and Genome Biology, Hospital for Sick Children, Toronto, Ontario M5G 0A4, Canada; 2 Department of Molecular Genetics, University of Toronto, Ontario M5S 1A8, Canada; 3 Department of Physiology, University of Arizona, Tuscon, Arizona 85724, USA

**Keywords:** Nemaline myopathy, NEBULIN, Modifier screen, Mice, Zebrafish

## Abstract

Nemaline myopathy is a rare neuromuscular disorder that affects 1 in 50,000 live births, with prevalence as high as 1 in 20,000 in certain populations. 13 genes have been linked to nemaline myopathy (NM), all of which are associated with the thin filament of the muscle sarcomere. Of the 13 associated genes, mutations in *NEBULIN* (*NEB*) accounts for up to 50% of all cases. Currently, the disease is incompletely understood and there are no available therapeutics for patients. To address this urgent need for effective treatments for patients affected by NM, we conducted a large scale chemical screen in a zebrafish model of *NEB*-related NM and an N-ethyl-N-nitrosourea (ENU)-based genetic screen in a mouse model of *NEB* exon 55 deletion, the most common *NEB* mutation in NM patients. Neither screen was able to identify a candidate for therapy development, highlighting the need to transition from conventional chemical therapeutics to gene-based therapies for the treatment of NM.

## Abstract (precis)

**Summary:** NEBULIN-related nemaline myopathy currently has no treatment. We attempted to uncover new avenues for therapy by performing modifier screens, which unfortunately failed to identify modifiers that improved disease relevant phenotypes.

## INTRODUCTION

Nemaline myopathy (NM) is a genetic muscle disorder characterized by severe muscle weakness and motor disabilities and defined by the pathognomonic appearance of nemaline rods on muscle biopsy (Darras, 2015; North and Ryan, 1993). Patients affected by NM are classified into six groups based on their age of onset and the severity of motor and respiratory disability: severe congenital NM (16% of the NM population), intermediate congenital NM (20%), typical congenital NM (46%), childhood or juvenile onset NM (13%), adult onset NM (4%) and other forms (such as the autoimmune disease termed sporadic late onset NM) (Ryan et al., 2001). Mutations in at least 13 genes can cause NM (Gonorazky et al., 2018), with recessive mutations in *NEBULIN* (*NEB*) representing the most common overall cause. *NEB* encodes a large 600–900 kDa protein responsible for regulating thin filament length and actin-myosin cross bridge dynamics (Labeit et al., 2011).

There are currently no therapies for NM. There is also a paucity of candidate treatments in the pre-clinical pipeline. A few potential therapies, such as L-tyrosine and taurine, have been identified based on pilot studies in small NM patient cohorts or via anecdotal off-label use by patients (Ryan et al., 2008). Recently, Bryson-Richardson and colleagues tested several of these supplements in a zebrafish model of *NEB*-related NM (Sztal et al., 2018). They determined that none of the molecules promoted improvement in muscle structure or function. Lack of effectiveness of L-tyrosine has also been observed in pre-clinical models of *ACTA1*-related NM (Messineo et al., 2018). These studies concluded that there is an urgent need to explore new strategies to identify and develop effective treatments for NM.

Establishing modifiers in model organisms may identify alternative therapeutic approaches. Chemical screens in zebrafish have uncovered a number of new drug targets in the treatment of cancers and genetic diseases (Gibbs et al., 2013b; Wiley et al., 2017). Due to the striking similarity in muscle structure and the conservation of key muscle-related gene products between fish and humans, zebrafish have proven to be an excellent model for muscle disease (Berger and Currie, 2012; Gibbs et al., 2013b). Importantly, zebrafish possess all known genes related to NM, and models of several of these genes have been established and characterized (Davidson et al., 2013; Gupta et al., 2013; Sztal et al., 2015; Telfer et al., 2012; Yuen et al., 2014). In particular, *neb* zebrafish mutants accurately model human NM (Sztal et al., 2015; Telfer et al., 2012): (1) recessive variants produce reduced/absent nebulin protein expression; (2) the muscle of *neb* mutants shows reduced thin filament length and the presence of nemaline bodies, two key pathologic features of the human disease; and (3) the overall phenotype is one of impaired movement, force generation and reduced survival. Of note, zebrafish have several properties (*ex utero* fertilization, rapid development, large offspring number) that make them ideal for large-scale chemical screens (Zon and Peterson, 2005). Screens have been performed in zebrafish models of other muscle diseases (Kawahara et al., 2011; Waugh et al., 2014; Widrick et al., 2019), but none have yet been performed in a model of NM.

Forward genetic modifier screens have proven successful in mice in uncovering targets for therapeutic intervention in previously untreatable disease (Buchovecky et al., 2013b; Hamilton and Yu, 2012; Philp et al., 2018). Successful demonstrations of forward screening approaches have been documented in mouse models of muscular dystrophy, primarily using a strategy of breeding the same mutation onto different genetic backgrounds. One prominent example of this approach was with a model of LGMD2C (due to SGCG mutation), where the second site modifier LTBP4 was identified (Heydemann et al., 2009). This has led to a new understanding of the pathogenesis of LGMD2C and related muscular dystrophies, and new avenues for therapy (Ceco et al., 2014; Flanigan et al., 2013).

A rapid strategy for applying forward genetics to identify modifiers in mice is to use the super mutagen N-ethyl-N-nitrosourea (ENU) (Geister et al., 2018). We have previously utilized an ENU-based genetic screen to uncover second site gene modifiers in a mouse model of Rett syndrome (Buchovecky et al., 2013a,b). To date, however, ENU mutagenesis modifier testing has yet to be applied to any mouse models of muscle disease. *NEB*-related NM is potentially well suited to modifier screening. There is evidence of phenotypic heterogeneity among individuals with *NEB* mutations that does not appear to be strictly dependent on the primary mutation, particularly in the patient group possessing our mutation of interest, the deletion of *NEB* exon 55 (Lehtokari et al., 2009, 2014, 2011). In addition, the neb protein interacts with several proteins and there is evidence that implicates signalling pathways as active modulators of neb’s function (Takano et al., 2010). Lastly, there is a mouse model of *NEB*-related NM with exon 55 deletion that has pathologic features consistent with NM and a severe and reproducible phenotype (death by age 7 days) (Ottenheijm et al., 2013).

The overarching goal of our work is to identify therapies for NEB-related NM. Currently there are few therapeutic targets, and limited strategies in the pre-clinical pipeline. Because of the lack of targeted strategies, we sought to use non-biased methodologies to identify new avenues for treatment. We pursued a large scale chemical screen in a zebrafish model of *NEB*-related NM, and an ENU-based genetic modifier screen in the *Neb* exon 55 deletion mouse. We did not identify any chemicals or genetic variants that positively modified the phenotype in these two models. While there are important caveats to any large-scale screen, our data suggest that *NEB*-related NM is challenging in terms of therapy development, and that strategies targeting the primary genetic cause may ultimately be most successful.

## RESULTS

### Drug screen in a zebrafish model of NEB-related NM

**Fig. 1.**
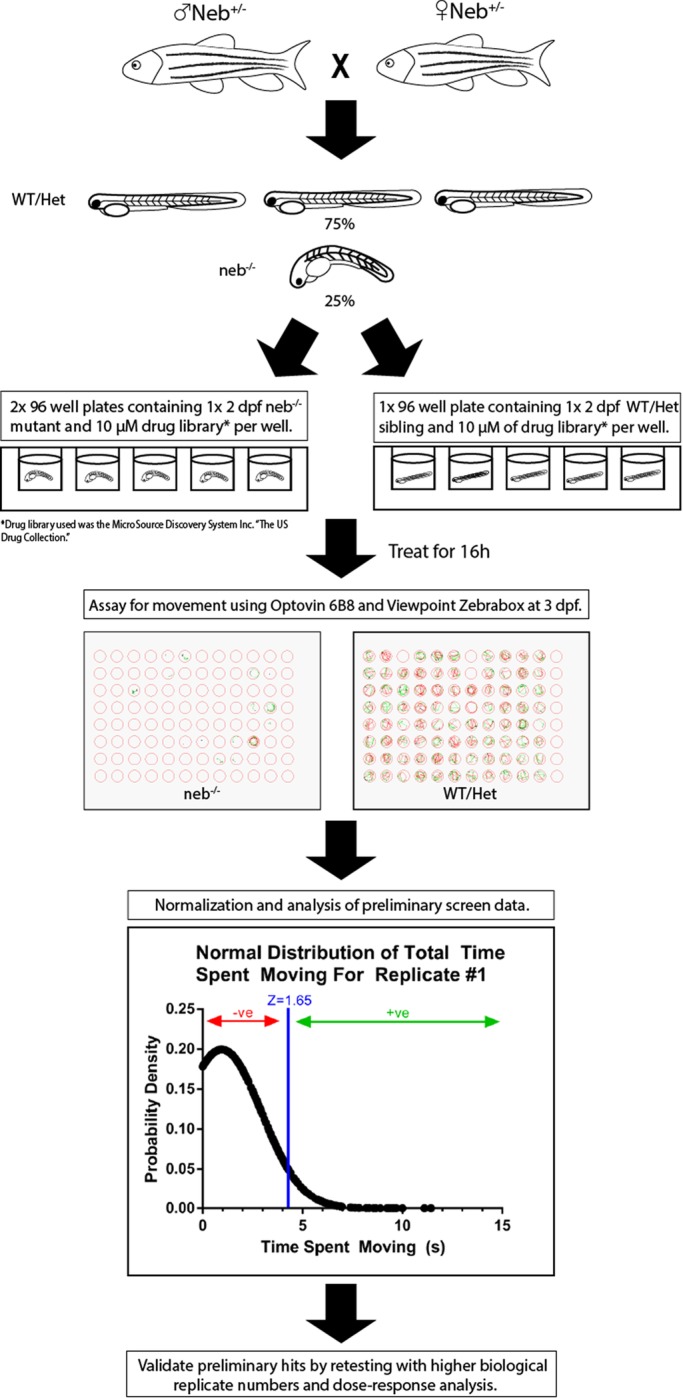
**Drug screen in a zebrafish model of NEB-related nemaline myopathy.** Schematic depicting the experimental flow of an unbiased drug screen in neb mutant zebrafish using the Microsource Discovery library. Heterozygous *neb* zebrafish (*neb*+/−) were mated, and *neb* mutant (*neb*−/−) offspring identified by phenotype. Once identified at 2 dpf, single *neb*−/− embryos were placed into individual wells of a 96-well plate, each containing a single drug at a dose of 10 μm. Embryos were incubated for 16 h, and then analyzed for movement using the Viewpoint Zebrabox. All drugs were tested in duplicate. Positive hits, as defined using a Z analysis of the movement data, were re-tested using *n*=16 neb (−/−) zebrafish and multiple doses of the drug. While eight drugs were initially identified as positive hits, no single compound gave a positive result upon retesting.

**Fig. 2.**
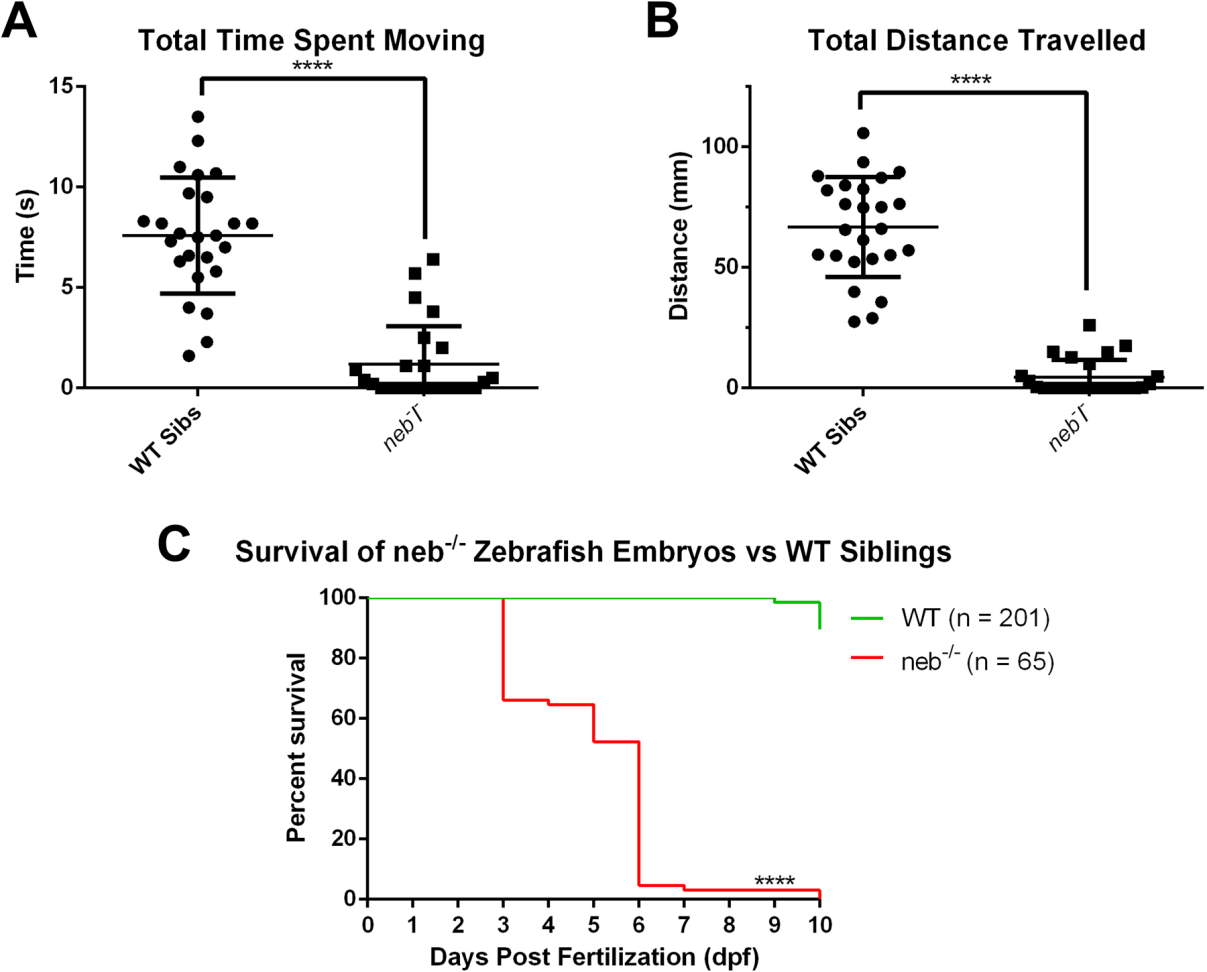
***neb^−/−^* embryos exhibit decreased mobility and survival relative to their WT siblings.** At 3 dpf, *neb^−/−^* embryos (*n*=25) displayed impaired movement both in terms of total time spent moving (A) and total distance travelled (B) relative to their wild-type (WT) siblings (*n*=25) when tested with the Viewpoint Zebrabox. (C) *neb^−/−^* embryos also exhibit dramatically lower viability relative to WT siblings, with most dying by 6 dpf. ****P<0.0001.

We performed a large-scale drug screen in our previously published zebrafish model of *NEB-*related NM (Fig. 1). This recessive model (originally obtained from the Sanger mutation resource) has a substitution mutation that impacts splicing of zebrafish *neb* exon 46 and results in loss of neb protein (Telfer et al., 2012). Embryos homozygous for the mutation (*neb*^−/−^ or ‘*neb*’) have a severe phenotype characterized by progressive lack of movement starting at 2 days post fertilization (dpf) and death by 7 dpf (Fig. 2). Using movement as our primary outcome, we tested 1360 compounds from a repurposing library (US Drug Collection Library, MicroSource Discovery Systems Inc.) composed of various compounds that have been approved by the FDA for use in clinical trials or commercial sale.

Briefly, 2 dpf *neb* zebrafish (identified by phenotype) were placed in individual wells of a 96-well plate, with each well containing a single drug at a concentration of 10 µM. This concentration was determined based on previous zebrafish screens using this library, including our own work with a model of Duchenne muscular dystrophy (Waugh et al., 2014). To identify positive ‘hits’ (i.e. chemicals that improved the *neb* movement phenotype), fish movement, as determined using the Viewpoint ZebraBox automated movement system, was then examined 24 h later. We then aggregated the movement data for all chemical compounds and normalized them to generate a normal distribution. Based on this, we established a Z-score for each of the chemical compounds tested.

We set our positive hit threshold at a Z-score of ≥1.65 and using this methodology, we identified eight preliminary compounds that demonstrated the potential to improve the mobility defect in *neb* zebrafish (*n*=2 screened per drug). Based on a theoretical power calculation performed using ZebraBox data from untreated *neb* 3 dpf embryos, testing of two embryos was predicted to be sufficient to detect a 50% increase in movement with significance.

We next took these eight preliminary compounds and re-tested them using identical screening methodology but now *n*=16 embryos. We tested a range of concentrations for each chemical compound (10, 20, 50 and 100 µM). However, with this re-testing, none of the eight compounds yielded a statistically significant change in movement as compared to untreated neb embryos examined in parallel. We thus were unable to confirm that any chemical compound was able to significantly improve the mobility of *neb* zebrafish.

### ENU-based suppressor screen in a mouse model of NEB-related NM

**Fig. 3.**
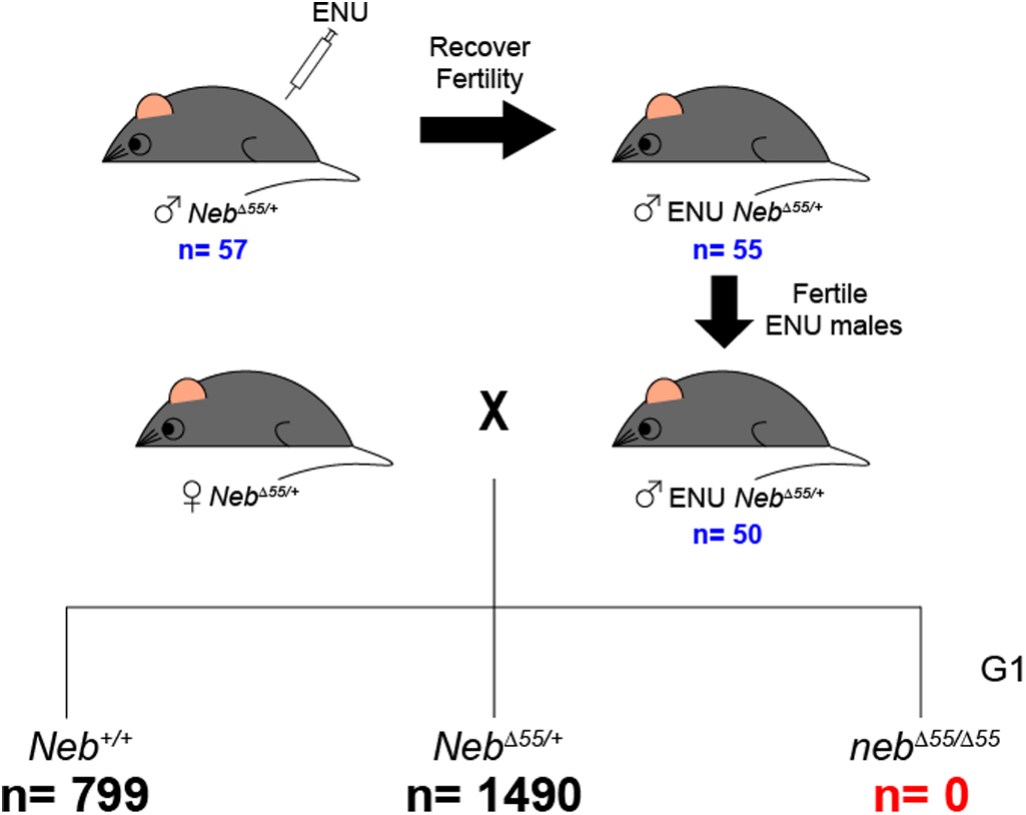
**ENU-based suppressor screen in a mouse** **model of NEB-related NM.** Schematic of dominant suppressor screen. Heterozygous male Neb mutant (*Neb*^Δ55/+^) mice were treated with ENU (90 mg/kg weekly for 3 weeks), then allowed to recover for up to 8 weeks, then tested for fertility by mating with female WT CD-1 animals. Fertile males were then mated on a rotating schedule with heterozygous Neb mutant (*Neb*^Δ55/+^) females. The resulting generation one (G1) offspring were monitored daily starting from birth. A subset of live-born pups exhibited an obvious phenotype consistent with the reported Neb phenotype. These animals all died by P7. Animals surviving beyond this point were monitored daily, and then genotyped by age 3 weeks. No animals surviving to 3 weeks had the Neb mutant genotype (*Neb*^Δ55/Δ55^). 14 G1 heterozygous mice unexpectedly had weakness (heterozygous mice are typically normal). However, upon inheritance testing the motor dysfunction phenotypes did not segregate with the heterozygous genotype. Of note, there was non-significant (*P*=0.0582) skewing of numbers of wild-type versus heterozygous mice, potentially indicating that some heterozygous mice had a second Neb mutation in trans that resulted in a perinatal lethal phenotype similar to that of *Neb*^Δ55^ homozygotes.

We also performed a dominant suppressor screen using the chemical supermutagen ENU in a mouse model of *NEB*-related NM (*Neb*^Δ55^) (Fig. 3). This previously characterized recessive model carries a deletion of *Neb* exon 55, which results in absent nebulin protein expression (Ottenheijm et al., 2013). Phenotypically, homozygous mice have small body size, impaired gait and die by postnatal day 7 (P7) (Ottenheijm et al., 2013). Similar to the human disease, mice heterozygous for the mutation (i.e. carriers) have no overt phenotype, can breed efficiently and have normal lifespan.

To identify potential suppressor mutations of the lethal phenotype, we injected 57 *Neb* heterozygous (*Neb*^Δ55/+^) male mice with ENU starting at 8 weeks of age (Buchovecky et al., 2013b). After ENU exposure, males were allowed to recover, and were subsequently mated to WT CD-1 mice to test fertility. 50/57 mice were confirmed as fertile and then used for subsequent suppressor screen. These 50 mice were mated to untreated *Neb*^Δ55/+^ females using a rotating breeding strategy. We screened the G1 offspring of these matings at 2–4 weeks of age for phenotypic suppression, with the hypothesis that a positive modifier would promote an increase of survival in *Neb*^Δ55^ mice at least to this age. In total, 2289 G1 mice were born, of which 799 were identified as WT and 1490 were heterozygous (*Neb^Δ55/+^*). We did not detect any mice at the age of screening that were homozygous for the *Neb* exon 55 deletion (*neb^Δ55^*). This indicates that no *neb^Δ55^* mice were able to survive until the 2-week mark, suggesting that no dominant suppressor gene variants were detected in our screen.

Overall, if the number of WT mice were equivalent in ratio to the number of homozygotes, we screened nearly one genome's equivalent for modifiers of *Neb*, since the mutation rate for ENU is one new mutation in every 700 genomes screened (Hitotsumachi et al., 1985). Despite this, we failed to identify a single homozygous mutant mouse that survived past P7. While our genomic screen did not approach saturation, our findings suggest that there may only a limited number of autosomal dominant mutations that are able to modify the NM phenotype in our mouse model. Of note, we did observe a skewing in favour of WT mice (O=799) versus heterozygous mice (O=1490, E=1598); however, this did not reach statistical significance (one-sided exact Binomial test, *P*=0.0582).

## DISCUSSION

There is a great unmet need for drug discovery and development for NM. In particular, despite the high burden of clinical disability in patients with NM and the presence of suitable animal models of disease, there are a paucity of targets in the pre-clinical pipeline. In an attempt to discover new potential therapeutic targets and pathways for NM, we performed two independent modifier screens in pre-clinical models of *NEB*-related NM. We failed to uncover evidence for improvement with either strategy, raising the possibility that *NEB*-related NM poses significant challenges in terms of drug discovery and identification of potential modifiers.

Our study represents the first large-scale screening efforts for *NEB*-related NM. The zebrafish screen was performed using methodology and dosing that we and others have previously established (Kawahara et al., 2011; Waugh et al., 2014; Zon and Peterson, 2005). In our previous successful screen using a DMD fish model we used birefringence as the primary outcome (Waugh et al., 2014), though we have successfully used movement (measured using the same methodology as described in the present screen) as an outcome for testing therapies in targeted screening in other models of related myopathies (Gibbs et al., 2013a; Robb et al., 2011; Sabha et al., 2016). The mouse screen was performed using a technical strategy that we advanced for studying a mouse model of Rett Syndrome (Buchovecky et al., 2013a,b). Our screen with the Rett Syndrome mouse used a similar number of breeding pairs, tested offspring number, and a G1 dominant modifier approach, and uncovered several genetic loci that modify the Rett mouse phenotype. We therefore approached both screens with confidence, and consider our negative results as informative in terms of defining the difficulty in identifying ‘second site’ treatments and modifiers for *NEB*.

There are important caveats to each screen. We performed our zebrafish drug screen at one standard dose (based on previous drug screens performed in several labs) (Kawahara et al., 2011; Rennekamp and Peterson, 2015; Waugh et al., 2014), so there is the potential for drug modifiers to exist that only work at either higher or lower concentrations. We also did not measure drug levels within individual fish due to the scale of our chemical screen and the technical challenge of measuring muscle exposure to drug in embryos that are microscopic in size and scale. We used only one measure of the *neb* phenotype (reduced movement), and tested drugs via a single time window (starting at 2 dpf and testing at 3 dpf). Lastly, our screen was confined to the known FDA-approved drug universe (as our library was an FDA repurposing library), and thus did not include novel chemicals from larger ‘drug-like’ libraries. Future directions for chemical screening-based drug discovery for NM thus could include testing of non-repurposing libraries and examining other phenotypes and dosing strategies. That said, our screen was performed using robust methodology on a disease-relevant outcome measure, and thus our failure to find a chemical modifier should be considered as a meaningful result.

In terms of the mouse suppressor screen, the biggest caveat is that we looked only for dominant modifiers. There is the potential that recessive variants in a modifier gene would promote improved survival. Also, while analyzing a substantial number of mice, we likely did not fully saturate the genome with our mutagenesis (i.e. we likely did not achieve a mutation in every gene in the genome), and thus we cannot be certain that a dominant modifier of NM does not exist in mice. It is also possible that the specific allele used in the mouse screen is recalcitrant to phenotypic modification. Future avenues in terms of genetic modifier screening could be to perform a recessive modifier screen, though this would require a great number of mice, additional mouse generations, and thus a much higher expense, or to try breeding the exon 55 deletion onto different genetic backgrounds (less expensive but takes 10 generations of back crossing and thus a long time frame).

Of note, one important challenge for any drug development approach for *NEB*-related NM is the lack of positive control for the experiments. For instance, there are no chemicals yet identified that are known to improve the movement phenotype or the reduced survival of *neb* zebrafish, and no known strategies for increasing survival of the exon 55 deletion mice.

Moving forward, there are some pathways, such as ubiquitination/protein turnover and actin-myosin cross bridge dynamics (de Winter et al., 2013; Ramirez-Martinez et al., 2017), that have been identified in models of other NM genetic subtypes that may yield targeted therapies for *NEB*-related NM. However, given our results and those of Bryson Richardson with a different *neb* zebrafish mutant, it may be most fruitful to focus therapy development on gene-based treatments. For example, nonsense mutations account for 23% of all *NEB* mutations reported in patients thus far (Lehtokari et al., 2014), and drugs that promote read through of premature stop codons may be viable candidates. Also, there are some recurrent *NEB* mutations, such as the exon 55 deletion (Lehtokari et al., 2009), which may be amenable to CRISPR-based gene editing.

## MATERIALS AND METHODS

### Zebrafish husbandry and lines

All zebrafish were housed, maintained and bred in accordance with Animal Use Protocols established by Animal Care Committee at PGCRL and the CCAC. *neb* zebrafish were obtained from the Zebrafish International Resource Centre (line hu2849). *neb* zebrafish were bred on an AB background (ZIRC).

### Genotyping

Genomic DNA samples were obtained through tail fin clips or whole embryos. Samples were then digested with Proteinase K (200 µg/ml) to extract the DNA. Extracted DNA was then used for genotyping using custom TaqMan SNP Genotyping probes (Thermo Fisher Scientific).

### Chemical screening

Adult *Neb^+/−^* zebrafish (hu2849, ZIRC) were crossed to produce *neb^−/−^* embryos. All embryos within the clutch were dechorinated at 1 dpf using pronase. *neb^−/−^* embryos were separated from their clutchmates at 2 dpf based on phenotype. At 2 dpf, *neb^−/−^* embryos were arrayed one per well into a 96-well plate containing 10 µM of various drugs from the US Drug Collection Library (MicroSource Discovery Systems, http://www.msdiscovery.com/usdrug.html). Embryos are treated for 16 h at 28.5°C. All compounds were tested on *n*=2 *neb^−/−^* embryos as calculated through statistical power analysis using the total distance (d) travelled data from untreated *neb^−/−^* embryos. Untreated 3 dpf neb−/− zebrafish travel 4.45 mm on average (*n*=25, s.d.=7.23) while untreated 3 dpf WT clutchmates travel 66.77 mm on average (*n*=25, s.d.=20.70). Based on these numbers, we set the positive hit threshold to be restoration of *neb^−/−^* embryo movement to 40% of WT levels (d=26.7 mm) and using α=0.05 and power=0.8, we determined that a minimum of *n*=2 *neb^−/−^* embryos was necessary to be tested per compound.

### Optovin 6B8 movement assay

Following treatment, embryos were screened for survival by visually detecting a heartbeat under a light microscope. To analyze gross movement, embryos were treated with 10 µM of optovin 6B8 (Hit2Lead) and incubated in the dark for 10 min. Following incubation, a Viewpoint Zebrabox machine (Viewpoint) was used to monitor zebrafish movement. Our Viewpoint protocol was: 10 s of dark, 10 s of light (100% strength) and 10 s of dark. Embryos were tested three times, with 2 min of rest between trials.

### Scoring of hits

Following completion of screening, movement data from all compounds tested were aggregated and normalized to give each compound a Z-score. Compounds with Z-score of ≥1.65 (*P*<0.05, one-tailed Z-test) were identified as potential positive hits. Z-scores were cross-referenced between our two replicate datasets to remove false-positives and drug compounds with Z≥1.65 for both duplicate trails were deemed our positive hits. Z≥1.65 corresponds to d=24.48 mm and 23.35 mm for our two replicate datasets.

### Secondary validation of hits

Positive hits were re-tested using *n*=16 *neb^−/−^* zebrafish and multiple doses of the drug (10 µM, 20 µM, 50 µM, 100 µM). While eight drugs were initially identified as positive hits, no single compound gave a positive result upon retesting.

### Mouse husbandry and strains

All mice were housed, maintained, bred and treated in accordance with the animal care and ethics protocols established by the Toronto Centre for Phenogenomics and CCAC. *neb^Δ55^* mice were obtained from our collaborators at the Granzier lab in the University of Arizona and maintained on a C57BL/6J background (The Jackson Laboratory).

### Genotyping

DNA was obtained from mice tail biopsies and digested in a 25 mM NaOH, 0.2 mM EDTA, pH 12 solution at 95°C. The digest solution was then neutralized with 40 mM Tris-HCl, pH 5 to produce PCR-ready DNA. PCR was conducted using three primers: WT forward, 5′-GCATTCTTGCTCTTTCTTGTATGG-3′; Δ55 forward, 5′-ACACGCGTCACCTTAATATGC-3′ and reverse, 5′-GAAAGGAACTCTGTCCTCTGG-3′, and visualized using a Qiagen QIAxcel Advanced system.

### ENU injections

57 *Neb^Δ55/+^* males were injected with ENU into the spermatogonial stem cells (90 mg/kg body weight, weekly for 3 weeks) starting at 8 weeks of age. Following injections, the mice were given 3 months to rest and recover from the injections. Fertility in injected males was tested through breeding to CD-1 females. 50/57 injected survived and recovered their fertility.

### Rotational breeding

Male ENU-injected *Neb^Δ55/+^* mice were bred on a rotational basis to maximize pup output before health deterioration due to ENU treatment. Males were bred to two uninjected female *Neb^Δ55/+^* mice for a week. Following a week, the males were then transferred to a new cage containing two alternate uninjected female *Neb^Δ55/+^* mice. This process was repeated on a 4-week schedule (i.e. four cages containing two female *Neb^Δ55/+^* mice per male, where each male spends 1 week per cage before rotating to the next). Our breeding strategy was approved by the Animal Care Committee (ACC) at the Toronto Centre for Phenogenomics. Each male was bred until a maximum of 100 pups was generated to prevent oversampling of a single genome.

### Ethics approval

All animal work was performed in accordance to international guidelines for the care, health, and safe-keeping of vertebrate animals. The zebrafish drug screen was performed under IACUC-approved protocol #41617 and the mouse ENU suppressor screen was performed under The Centre for Phenogenomics-approved protocol #21-0319H.
